# Influence of stacking sequence on the mechanical properties of banana-glass fiber hybrid laminates for automotive shells

**DOI:** 10.1016/j.heliyon.2024.e40130

**Published:** 2024-11-06

**Authors:** Mohammad Kamruzzaman, Samiul Alam

**Affiliations:** Department of Mechanical Engineering, Khulna University of Engineering & Technology, Khulna, 9203, Bangladesh

**Keywords:** Natural fiber, Hybrid laminated composite, Automotive shell

## Abstract

The development of composite materials using natural fibers has garnered significant global research interest. Banana fibers, due to their abundant availability and rapid growth cycle, present a promising option for composite material development. When combined with synthetic fibers to form hybrid composites, the resulting material may exhibit significantly improved properties, which is desirable for automotive body manufacturing. The hybrid properties of such composites, however, remain largely unexplored and require thorough evaluation. This study investigates the mechanical performance of hybrid laminated composites fabricated from banana and woven glass fibers bonded with epoxy resin. The composites were produced using non-crimp banana (B) fiber and woven glass (WG) fiber fabrics through the hand lay-up process with four distinct stacking sequences: [WG/B-0⁰/B-0⁰/WG], [WG/B-0⁰/B-45⁰/WG], [WG/B-0⁰/B-60⁰/WG], and [WG/B-0⁰/B-90⁰/WG]. Comprehensive mechanical testing, including tensile, flexural, impact, and hardness tests, along with environmental testing (water diffusion) was conducted in accordance with ASTM standards. Results indicated that the [WG/B-0⁰/B-0⁰/WG] configuration exhibited the highest tensile strength, while the [WG/B-0⁰/B-60⁰/WG] configuration achieved superior flexural strength. The [WG/B-0⁰/B-45⁰/WG] configuration demonstrated the highest impact strength and energy absorption. Rockwell hardness testing revealed that the average hardness numbers for all orientations were approximately 79, which is considered moderate for composite materials. Water absorption tests showed maximum water saturation in [WG/B-0⁰/B-90⁰/WG] of ∼5.5 %, where the other laminates had water saturation between 4.5 and 4.9 %. The microscopic analysis of the [WG/B-0⁰/B-45⁰/WG] laminate suggests fiber-matrix debonding and fiber pull-out as the major cause of failure under tensile and flexural loading. These findings suggest that the hybrid laminated composites, particularly the [WG/B-0⁰/B-0⁰/WG] and [WG/B-0⁰/B-45⁰/WG] configurations, exhibit mechanical properties suitable for automotive body applications.

## Highlights

• Hybrid composites with banana/glass fibers showed promise for automotive bodies.
• Different stacking sequences were evaluated through mechanical testing.
• Water absorption and failure mechanisms were analyzed for real-world automotive use.
• Optimal orientation was suggested, and future research scope was proposed for further study.

## Graphical abstract

**Figure:**
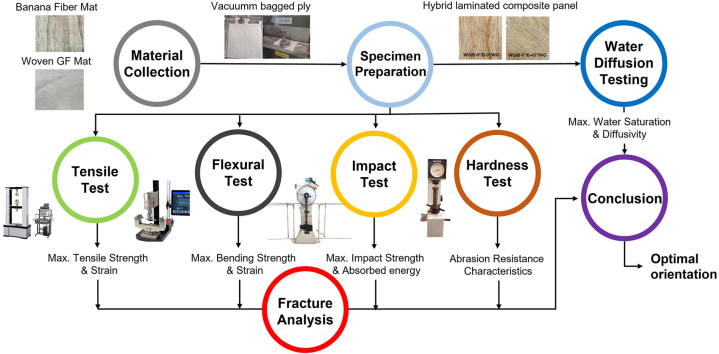

## 1 Introduction

In recent years, natural fibers have been employed as composite fiber reinforcement in a variety of applications such as aerospace [1], marine [2], sporting goods [3], and automotive industries [4]. Researchers, scientists, engineers, and professionals worldwide have been fascinated by the distinctive qualities and properties of natural fiber [5]. The outstanding properties of the fiber reinforced polymer (FRP) composite can be primarily attributed to the reinforcing fibers, which are often significantly stronger than the matrix [6]. Hybrid composites, which integrate both synthetic and natural fibers, have emerged as a feasible solution for automotive applications. These composites have the potential to enhance material characteristics, decrease weight, and enhance environmental sustainability in comparison to conventional materials such as steel and aluminum [7].

In general, natural fibers possess several advantages over synthetic fibers [8]. These include their low density, eco-friendliness, lack of toxicity, renewability, bio-standardization, relative non-abrasiveness, high specific strength and modulus, as well as ease of processing. Although natural fibers offer numerous benefits, they also have a few drawbacks including limited strength, low modulus, high moisture absorption, and susceptibility to flammability [7]. Hydrophilic and moisture-absorbing natural fibers face several issues, one of which is the absence of strong bonding between the fibers and the surrounding matrix [9]. In addition to the fiber's hydrophilic nature, common polymer matrices (e.g., epoxy, acrylonitrile-butadiene-styrene) are also significantly influenced by long-term water diffusion [10,11]. The high moisture sensitivity of conventional fibers in composites, leading to a decrease in mechanical properties and delamination, poses a significant issue when utilizing them in these materials [12]. The loss in mechanical qualities may be attributed to inadequate interfacial adhesion between fibers and matrix [13]. In order to address these drawbacks, the hybridization approach is employed.

Using synthetic fiber for composite reinforcement may leave significant detrimental impact on environment in long term application, where partial substitution through natural fibers may be an effective solution to this issue. Contemporary composite materials engineering is currently investigating the environmental and physical characteristics of glass fiber when coupled with organic constituents like hemp [14], jute [[15], [16], [17], [18]], wood [19], flax [20,21], sisal [22], basalt [23], and banana [24]. *Musa cavendishi* and *Musa sapendum* are banana plant varieties that yield edible fruits and are also utilized for the extraction of banana fiber [25]. Most of the fiber comes from the plant's stem. Around 37 kg of stem yield roughly 1 kg of good-quality fiber. Natural fibers are particularly efficient in providing insulation against sound, heat, and electricity, even at elevated temperatures and low ash content in pyrolysis [26,27]. It exhibits good endurance, with elevated levels of resistance to deformation under constant stress and toughness.

There are several studies based on the concept of combining synthetic and natural fibers with polymer matrix composites through hybridization for automobile application [28]. The incorporation of coconut coir with glass fiber in a composite material significantly expanded the potential applications of hybrid composites in several fields of engineering and technology. The mechanical properties, including tensile strength, hardness, and impact strength, were assessed on sections of the composite material composed of natural fibers that are beneficial for applications in automotive and aerospace engineering [28]. Macroscopic investigations were conducted to examine the interfacial characteristics of the laminate [[29], [30], [31], [32]]. The mechanical properties of composites are greatly affected by the orientation of the fibers. Jute fiber composites exhibited enhanced flexural strength when arranged in unidirectional and cross-ply orientations (e.g., 0/0/0/0 and 0/+45°/-45°), indicating that appropriate alignment can improve the material's ability to withstand bending stress [33]. The significance of orientation also applies to glass/epoxy composites, the flexural strength was significantly higher in the (0/90°) orientations compared to the (0/45°) orientations [34] In some cases, thicker composites exhibited improved tensile strength. Composites that are oriented at a 30° angle have the highest tensile strength when the thickness is raised [35]. The total mechanical performance of biphenyl matrix composites was significantly influenced by the orientation of the fibers [36]. Similarly, the tensile and compressive properties of hybrid composites consisting of glass and satin fabric with epoxy matrices were greatly influenced by the variable fiber quantity and orientation [37]. Experimental results demonstrate that the alignment of fibers in coconut midrib epoxy composites had a significant impact on hardness, flexural strength, and impact resistance [38]. These studies collectively indicated that in order to attain the best mechanical qualities, it is necessary to take into account aspects such as fiber orientation and material thickness. Polymeric composites can experience mechanical damage due to compression, tension, and bending. Therefore, it is imperative to have high-scale damage tolerance and good mechanical properties. The use of a strong matrix, support with woven textures in two directions, and sewing of fibers in epoxy-based polymer composites increases damage tolerance [[39], [40], [41]]. Hybrid composites are an advancement in development when compared to standard FRP composites. Reinforced composites provide superior flexibility and durability compared to other alternatives. The mechanical properties can vary depending on the different orientations of fibers or plies, as well as the varied volume fractions of fibers [42].

To assess the suitability of composite panels for automobile body or shell applications, it is imperative to conduct a series of tests, including tensile, flexural, impact, hardness, and water diffusion tests. These tests are essential for evaluating the material's performance and determining its compatibility with automotive applications. The implementation of this thorough evaluation process is essential for the creation of composite panels that exhibit not just safety and dependability, but also the capacity to function optimally during the whole lifespan of the vehicle. While several studies have explored hybrid composites of natural and synthetic fibers, this research is novel in its focus on banana fibers, which are abundant but underutilized in high-performance applications such as automotive shells. Moreover, the stacking sequence optimization specifically tailored for automotive performance has not been extensively addressed in the literature. Our study not only evaluates mechanical properties but also introduces environmental performance (water diffusion) as a critical aspect in the hybridization of banana and woven glass fibers for automotive applications. The combination of these factors—fiber type, orientation, mechanical and environmental testing—provides a new perspective on hybrid composites tailored for automotive applications. This research aims to make a significant contribution to the advancement of hybrid composites that leverage the advantages of both natural and synthetic fibers, providing a long-lasting and effective solution that is specifically tailored to meet the needs of the automobile industry.

## 2 Material and methods

### 2.1 Specimen fabrication

**Fig. 1:**
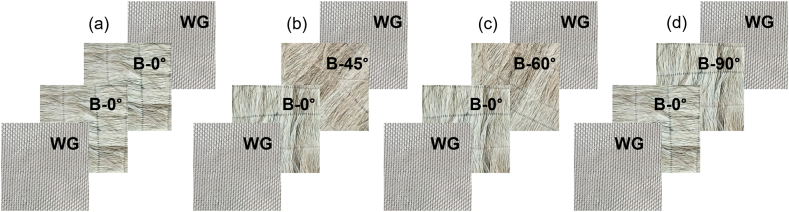
Differently oriented hybrid laminate demonstration for (a) [WG/B-0⁰/B-0⁰/WG], (b) [WG/B-0⁰/B-45⁰/WG], (c) [WG/B-0⁰/B-60⁰/WG], and (d) [WG/B-0⁰/B-90⁰/WG].

The banana-glass fiber hybrid laminated composite consisted of differently oriented unidirectional (UD) non-crimp fabric of banana (B) fiber, woven glass fiber (WG) fabric and commercially available epoxy resin. This hybrid composite is a 4-ply structure, with the top (1) and bottom (4) plies consisting of WG fabric and the middle (2 and 3) plies composed of banana fiber mats. The specific stacking sequences investigated were [WG/B-0⁰/B-0⁰/WG], [WG/B-0⁰/B-45⁰/WG], [WG/B-0⁰/B-60⁰/WG], and [WG/B-0⁰/B-90⁰/WG]. The raw banana fibers were sourced from local distributors in Khulna, Bangladesh which was later processed to produce long strands of fiber. These fibers were laid up and sewn together in through-the-thickness direction (out of plane/z-direction) to form the UD non-crimp fabrics. The 45⁰ and 60⁰ plies were later cut from the baseline UD fabrics. Finally, the plies were cut to a square of 300 mm × 300 mm and a uniform thickness of 1 mm was maintained. The plain weave woven glass fabrics were purchased from Changzhou Right Composite Co. ltd, China. The WG fabrics were cut to match the dimensions of the banana fiber mats, the thickness of these plies was 0.15 mm. The matrix material was Little Sparrow 318AB-9T, a two-part epoxy (part A-epoxy, part B- curing agent/hardener) acquired from Glaros BD Limited, Bangladesh. To achieve an optimum property, a stoichiometric ratio of 0.93 was maintained for the epoxy cure, which led the mixing ratio between epoxy and the curing agent to be 10:1 by volume. The composite was manufactured through a hand lay-up procedure and cured in an oven with vacuum bagging. The plies were stacked in the aforementioned sequences and Fig. 1 depicts a visual representation of the stacking of the plies. The laminate was vacuum bagged with a −1 bar pressure and cured in an oven at 100 °C for 2 h. External pressure of 15 psi was applied by weight blocks while the vacuum pressure was maintained throughout the curing cycle. The layup was slowly cooled overnight inside the oven and taken out of the vacuum bag after 24 h. The final laminates had a varying thickness and density based on orientation as listed in Table 1. The fiber volume fraction (V_f_) was maintained at 30 %, which contained approximately 15 % glass fiber and 85 % banana fiber.

**Table 1:** Summary of the test methods and specimen geometry for differently oriented laminates.

Specimen No.	Specimen Thickness (mm)	Density (gcm^−3^)	Tensile Test (ASTM D3039) mm × mm	Flexural Test (ASTM D790) mm × mm	Impact Test (ASTM D256) mm × mm	Hardness Test (ASTM E18) mm × mm	Water Absorption Test (ASTM D570) mm × mm
[WG/B-0⁰/B-0⁰/WG]	5.1	0.96	250 × 25	196 × 13	65 × 10	10 × 10	30 × 30
[WG/B-0⁰/B-45⁰/WG]	5.2	2.2		200 × 13			
[WG/B-0⁰/B-60⁰/WG]	4	0.84		154 × 13			
[WG/B-0⁰/B-90⁰/WG]	4.2	1.12		161 × 13			

### 2.2 Mechanical test methods

To assess the mechanical performance of the hybrid laminated composites under conditions representative of automotive shell applications, a series of tensile, flexural, impact, and hardness tests were conducted. All tests were performed in accordance with relevant ASTM standards to ensure the reliability and comparability of results. Table 1 represents a summary of the test methods and specimen geometry of differently oriented laminates.

#### 2.2.1 Tensile testing

Tensile tests were conducted using a computer-controlled universal testing machine, following ASTM D3039 [43]. Specimens were prepared by cutting 250 mm × 25 mm samples from the composite laminates using water jet cutting, which ensures precision and prevents thermal damage to the material. The edges were meticulously sanded to remove any notch-like defects that could act as stress concentrators during loading. The tensile tests were performed in a displacement-controlled manner at a constant strain rate of 5 mm/min. During the test, an extensometer was attached to the specimen to accurately measure the strain. The test continued until specimen failed.

#### 2.2.2 Flexural testing

Flexural properties were evaluated using a three-point bending test, adhering to ASTM D790 [44]. The span-to-thickness ratio was maintained at 32:1, with a standard specimen width of 13 mm. The test was performed on a universal testing machine equipped with a three-point bending fixture. The loading nose applied force at the midpoint of the specimen while the supports were positioned at the calculated span length. The test was conducted at a crosshead speed of 2 mm/min, and the force-deflection data were recorded until specimen failure.

#### 2.2.3 Impact testing

The impact resistance of the composites was assessed using a Charpy impact test, following ASTM D256 [45]. Specimens were prepared with dimensions of 65 mm × 10 mm, and a V-notch with a 45° angle was precisely machined at the midpoint of each specimen using a specialized notching machine. The test was conducted using a pendulum-type impact tester, where the notched specimen was placed horizontally and impacted by a swinging pendulum. The energy absorbed by the specimen during fracture was measured, which provides insight into the material's toughness and its ability to withstand sudden impacts—a critical consideration for automotive applications where crashworthiness is essential.

#### 2.2.4 Hardness testing

Rockwell hardness testing was conducted in accordance with ASTM E18 [46]. Specimens were prepared with dimensions of 10 mm × 10 mm and carefully polished to achieve a smooth surface finish, ensuring accurate indentation readings. The test was performed using a Rockwell hardness tester with a diamond indenter. The indenter applied a preliminary minor load followed by a major load, with the difference in depth of indentation used to calculate the Rockwell hardness number (HR). The test was repeated at multiple locations on each specimen to ensure consistent results. The hardness number reflects the material's resistance to indentation and provides insights into its surface durability, particularly in environments where abrasion and contact wear are factors.

### 2.3 Environmental testing method

Water diffusion has a major impact on the mechanical performance of fiber reinforced polymer composites. The mechanical cohesiveness of the chains is reduced as a result of water molecules breaking the secondary bonds that bind the macro-molecular polymer networks together. This causes additive loss, material swelling/thinning, and microcrack formation in addition to decreasing the glass transition temperature (T_g_). Water diffusion tests were conducted on 30 mm × 30 mm samples from each laminate to evaluate the performance of the hybrid laminated composite panels. Diffusion tests were performed with specimens submerged in deionized water at room temperature until three successive weight measurements showed an average mass gain in water of no more than 1 % or 5 mg, whichever value was greater, in accordance with ASTM D570 [47]. The specimens were periodically withdrawn and cleaned using dry paper towels, and their weights were recorded during the testing. The change in weight (*M*_*t*_) of the specimens due to water diffusion can be determined using Eqn. (1).

[1] $$M_{t}=\left(W_{t}-W_{0}\right)\times 100\%\;/\;W_{0}$$

where *W*_*t*_ is the wet weight at time *t*, and *W*_0_ is the dry weight.

## 3 Results

### 3.1 Mechanical test

The tensile and flexural strengths of the hybrid laminated composites are influenced by factors such as fiber orientation, the number of layers, temperature, and surface flaws. In this study, all samples were fabricated with a consistent V_f_ = 30 % and conducted at a constant temperature of 22 °C which ensured that variations in the mechanical properties could be attributed primarily to differences in fiber orientation and internal microstructure.

**Fig. 2:**
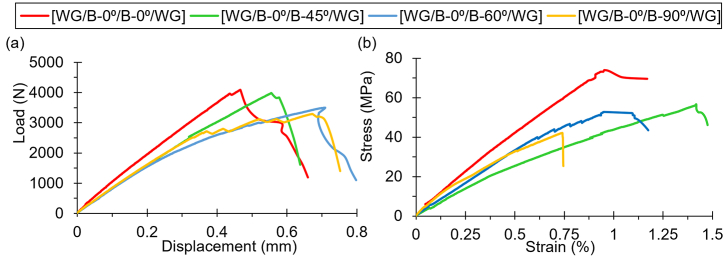
Comparison of (a) load against displacement and (b) stress against strain under tensile loading for differently oriented laminates.

Tensile tests revealed that the force-displacement behavior of all specimens followed a similar pattern: a linear increase in force with displacement until crack initiation, after which the crack propagated with little additional load. The tensile strengths across different fiber orientations showed distinct trends. The [WG/B-0⁰/B-0⁰/WG] orientation demonstrated the highest tensile strength, reaching a maximum stress of 72.48 MPa at 0.97 % strain, indicating that this configuration is more resistant to tensile forces compared to others. This was followed by the [WG/B-0⁰/B-45⁰/WG] and [WG/B-0⁰/B-60⁰/WG] orientations, while the [WG/B-0⁰/B-90⁰/WG] orientation exhibited the lowest tensile strength. [WG/B-0⁰/B-45⁰/WG] orientation reached a tensile strength of 55.58 MPa at 1.42 % strain, while the [WG/B-0⁰/B-60⁰/WG] orientation achieved 50.63 MPa at 1.11 % strain. The [WG/B-0⁰/B-90⁰/WG] orientation displayed the tensile strength at 42.73 MPa with a strain of 0.67 %. Moreover, variations in stress-strain behavior were noted among the orientations. The [WG/B-0⁰/B-0⁰/WG] orientation withstood the highest stress but also displayed a low strain, indicating a combination of strength and brittle nature. On the other hand, the [WG/B-0⁰/B-90⁰/WG] orientation, while having the lowest tensile strength, also showed less strain, suggesting a more brittle failure mode. This trend suggests that aligning the banana fiber mats at 0° relative to the woven glass layers enhances tensile performance, possibly due to an optimized load transfer between the fibers and the matrix and relatively more fiber allocation in the loading direction. However, with a little low strength but much higher strain than [WG/B-0⁰/B-0⁰/WG], [WG/B-0⁰/B-45⁰/WG] orientation showed a balanced combination of strength and moderate ductility, making it more resistant to brittle failure compared to other configurations. Fig. 2 depicts the load vs displacement and stress vs strain characteristics under tensile loading for differently oriented laminates.

**Fig. 3:**
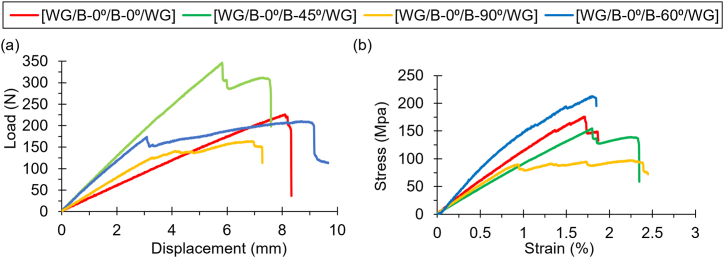
Comparison of (a) load against displacement and (b) stress against strain under flexural loading for differently oriented laminates.

In flexural tests, the performance trend differed from that observed in tensile tests. The [WG/B-0⁰/B-60⁰/WG] orientation excelled in flexural strength, with a maximum stress of 210.29 MPa at 1.77 % strain. This suggests that the alignment of fibers at 60° provides superior resistance to bending loads, making this configuration ideal for applications where flexural strength is critical. The [WG/B-0⁰/B-0⁰/WG] orientation, which performed best in tensile tests, showed a slightly lower flexural strength of 167.31 MPa at 1.63 % strain. The [WG/B-0⁰/B-45⁰/WG] orientation, despite its lower tensile strength, performed well in flexural tests, reaching 150.45 MPa at 1.96 % strain. This indicates that the 45° orientation might be more suitable for applications where resistance to bending is crucial, even though its tensile performance is lower. The [WG/B-0⁰/B-90⁰/WG] orientation, with a flexural strength of 97.63 MPa at 2.63 % strain, once again showed the least strength, underscoring its limited suitability for load-bearing applications. Fig. 3 depicts the load vs displacement and stress vs strain characteristics for differently oriented laminates under flexural loading. Table 2 provides the summarized results for tensile and flexural tests.

**Table 2:** Summarized result of tensile and flexural test for differently oriented laminates.

Orientation	Tensile Test				Flexural Test			
	Peak Load (N)	Max Stress (MPa)	Max Strain (%)	Elastic Modulus (GPa)	Peak Load (N)	Max Stress (MPa)	Max Strain (%)	Flexural Modulus (GPa)
[WG/B-0⁰/B-0⁰/WG]	4169.1	72.5	0.97	7.1	235.5	167.3	1.63	10.5
[WG/B-0⁰/B-45⁰/WG]	3982.8	55.6	1.42	6.8	340.6	150.5	1.96	12
[WG/B-0⁰/B-60⁰/WG]	3499.3	50.6	1.11	6.5	226.1	210.3	1.77	11.9
[WG/B-0⁰/B-90⁰/WG]	3290.3	42.7	0.67	4.5	162.2	97.6	2.63	7.5

**Fig. 4:**
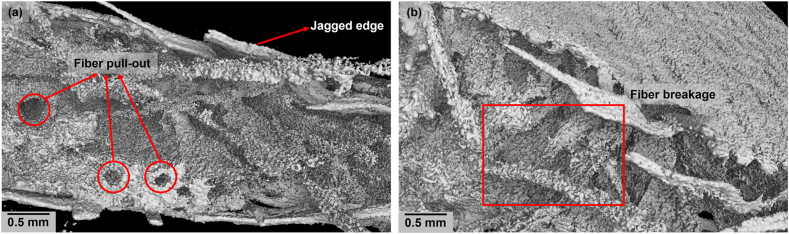
Fracture pattern analysis of [WG/B-0⁰/B-45⁰/WG] laminate under (a) tensile loading failure and (b) flexural loading failure.

The fracture patterns in both tensile and flexural tests suggest that all specimens primarily exhibited brittle failure, characterized by rapid crack propagation after reaching maximum load. However, the [WG/B-0⁰/B-45⁰/WG] orientation displayed a more gradual failure in tensile and flexural tests, with the crack propagation occurring over a longer strain range. While this orientation reached the moderate tensile and bending stress, it allowed for more deformation before failure, which could be beneficial in applications where energy absorption is important. Considering the balanced performance of [WG/B-0⁰/B-45⁰/WG] laminate in both tests, fracture analysis was performed only on this laminate. The microscopic view of the fracture surface of the [WG/B-0⁰/B-45⁰/WG] laminate (Fig. 4a) under tensile testing highlights several critical features: fiber pull-out, jagged edges, visible matrix cracking, and delamination. Fiber pull-out suggests that the interfacial bond between the fibers and the matrix was not sufficiently strong, leading to a common failure mode in composites where fibers disengage from the matrix rather than breaking. The jagged and rough fracture edges indicate a brittle failure, which occurs when the material abruptly breaks without significant prior deformation. Matrix cracking observed in the sample further confirms that the applied tensile stress exceeded the matrix's capacity, resulting in its rupture. Lastly, the presence of delamination, where the layers of the laminate have separated, underscores a critical failure mode in layered composites, often due to weak interlayer bonding or uneven stress distribution. The microscopic view of the fracture surface of the [WG/B-0⁰/B-45⁰/WG] laminate (Fig. 4b) under a flexural test highlights several critical features. The varied fiber orientations (0⁰ and 45⁰) contribute to complex fracture patterns, with 45⁰ fibers creating more intricate paths compared to the 0⁰ fibers. Matrix cracking is evident, typically originating from the tension side due to bending stress. Delamination is also observed, particularly at the interfaces between different fiber orientations, indicating separation of layers. Additionally, fibers show signs of both pull-out and breakage, with 45⁰ fibers more prone to pull-out due to the shear stresses experienced during bending.

In the impact test, as pendulum hammer stroke the composite specimen from a predefined height, it simulated the kind of impact that automotive shells might experience. Upon impact, the crack propagated through the fiber and matrix, initially severing the fibers and causing catastrophic material failure. As the fracture progressed, it shifted direction, running parallel to the fibers in the matrix, which significantly increased the test surface area. This larger fracture area allowed for more energy absorption due to the debonding between the fibers and the matrix. The initial strike by the pendulum initiates crack propagation, but not before the fibers absorb a portion of the energy. This energy absorption contributes to the composite's impact resistance, which is notably higher in certain fiber orientations. Among the tested orientations, the [WG/B-0⁰/B-45⁰/WG] configuration demonstrated the highest impact strength at 408.75 kJ/m^2^, reflecting its superior energy absorption due to the angular placement of the banana fiber mat. This is followed by the [WG/B-0⁰/B-60⁰/WG] orientation, which showed a slight decrease in impact strength to 384.23 kJ/m^2^, still higher than the [WG/B-0⁰/B-0⁰/WG] and [WG/B-0⁰/B-90⁰/WG] orientations, which exhibited impact strengths of 327 kJ/m^2^ and 335.18 kJ/m^2^, respectively. In terms of energy absorbed during impact at ∼200J, the [WG/B-0⁰/B-45⁰/WG] orientation again performed best, absorbing 16.35 J, while the [WG/B-0⁰/B-60⁰/WG] orientation absorbed 15.37 J. The [WG/B-0⁰/B-0⁰/WG] and [WG/B-0⁰/B-90⁰/WG] orientations absorbed 13.08 J and 13.41 J, respectively. These results highlight that while thickness and fiber volume fraction play crucial roles in determining a composite's impact strength, the orientation of the fibers within the laminate is also a significant factor. The findings suggest that hybrid composites with specific fiber orientations can exhibit enhanced impact resistance, making them potentially suitable for applications requiring materials that can withstand significant impact forces, such as automotive body panels.

**Fig. 5:**
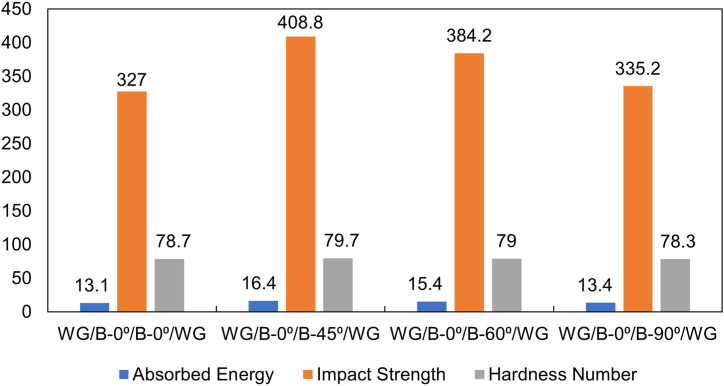
Comparison between impact energy, impact strength and hardness number for all orientations.

Hardness testing is a critical measure of a material's ability to resist localized surface deformation, often associated with its durability and wear resistance. The hardness of a composite is determined using a small indenter, which creates an indentation on the surface. A smaller indentation indicates a higher hardness value, reflecting greater resistance to surface damage. The maximum hardness was observed in the [WG/B-0⁰/B-45⁰/WG] orientation, with the [WG/B-0⁰/B-60⁰/WG] orientation following closely behind. The variations in fiber orientation significantly influence the hardness of the composite surface. Specifically, the [WG/B-0⁰/B-45⁰/WG] and [WG/B-0⁰/B-60⁰/WG] orientations feature different inner surface alignments, leading to increased hardness values in these configurations. This suggests that specific fiber orientations, particularly those with angular deviations from the 0⁰ alignment, enhance the composite's resistance to surface indentation. Fig. 5 portrays the comparison among impact energy, impact strength and hardness number for the laminates.

### 3.2 Water diffusion test

**Fig. 6:**
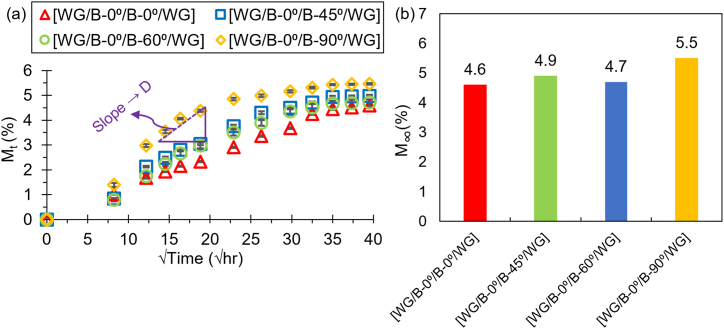
(a) Specimen weight change *M*_*t*_ (%) over time and (b) maximum water saturation level *M*_*∞*_ (%) of hybrid laminates.

Fig. 6a and b illustrate the time evolution of water absorption (*M*_*t*_ in Eqn. (1)) and the maximum water saturation levels (*M*_*∞*_) for the hybrid laminates over a period of approximately 65 days. The weight gain of the specimens due to water diffusion was recorded and plotted against the square root of time—a common method used to estimate the diffusion coefficient from the slope of the linear portion of the graph. For reference, ∼40 h on the x-axis of Fig. 6a corresponds to approximately ∼1600 h in the actual diffusion experiment. The Fickian diffusion model [48], which explains mass transport driven by concentration gradients, fits well with the observed trends. In this model, water molecules diffuse through the material at a rate proportional to the concentration gradient. Initially, water absorption increases linearly (indicative of the diffusivity of the material) before reaching saturation, where the material can no longer absorb additional water.

Water diffusion in fiber-reinforced composites is a complex process influenced by several factors, including fiber orientation, matrix composition, and the interface between fibers and the matrix. In this study, water diffusion occurred primarily through three pathways: (1) diffusion through the polymer matrix, (2) capillary action along the fiber-matrix interface, and (3) ingress into micro-voids and cracks within the composite structure. These mechanisms are more pronounced in fiber orientations where fibers are perpendicular to the water diffusion path, such as in the [WG/B-0°/B-90°/WG] configuration. The perpendicular orientation creates additional capillary pathways for water molecules, leading to increased water absorption. The water absorption trends across all laminates were quite similar, indicating that fiber orientation had minimal impact on the overall diffusion characteristics. However, a closer analysis revealed that the [WG/B-0°/B-90°/WG] laminate exhibited a slightly higher water absorption rate, absorbing around 5.5 % of water by weight, compared to the other laminates, which absorbed approximately 4.6–4.9 %.

From the slope of the linear portion of the absorption curves, the diffusion coefficients (*D*) were calculated for each laminate orientation. The [WG/B-0°/B-90°/WG] laminate had the highest diffusivity at 0.0097 mm^2^/h, consistent with its higher water absorption. In contrast, the [WG/B-0°/B-0°/WG] laminate had the lowest diffusivity at 0.0027 mm^2^/h, reflecting its relatively lower water uptake. The [WG/B-0°/B-45°/WG] and [WG/B-0°/B-60°/WG] laminates had diffusivities of 0.005 mm^2^/h and 0.0046 mm^2^/h, respectively, showing intermediate behavior between the 0° and 90° laminates. Overall, while all laminates exhibited similar trends in water absorption, the fiber orientation had a noticeable effect on both the rate of water diffusion and the total amount of water absorbed. The 90° laminate's higher water uptake and diffusivity suggests that it may be less suitable for applications requiring minimal water absorption, whereas the 0° laminate may offer better resistance to water ingress.

The hybrid banana-glass fiber composites developed in this study have promising applications in the automotive industry, particularly for body panels and shell structures, where lightweight and moderate strength are essential. The improved tensile, flexural, and environmental resistance properties make these materials suitable for components exposed to moisture and outdoor conditions. Looking ahead, future research could explore additional fiber orientations, such as [WG/B-0⁰/B-22.5⁰/WG], to further optimize performance. Enhancements in fiber-matrix bonding through chemical treatments or surface modifications could also improve durability, particularly in moisture-prone environments. Advanced manufacturing techniques, such as additive manufacturing or automated fiber placement, may offer greater precision in tailoring fiber orientations. These advancements, combined with ongoing efforts in sustainability and recyclability, could broaden the use of hybrid composites beyond the automotive sector, extending into aerospace, marine, and construction industries.

## 4 Conclusion

This research evaluated the performance of hybrid composites made of woven glass (WG) and banana (B) fibers under varying fiber orientations, focusing on their mechanical properties and potential applications in automotive components. The results demonstrate that hybridization of natural and synthetic fibers can effectively enhance the mechanical properties of composites while mitigating some of the inherent drawbacks of natural fibers, such as low strength and high moisture absorption. The [WG/B-0⁰/B-0⁰/WG] and [WG/B-0⁰/B-45⁰/WG] orientations showed the most potential for automotive application based on the conducted mechanical and environmental tests. Microscopic analyses of the fracture surfaces of [WG/B-0⁰/B-45⁰/WG] laminate under tensile and flexural tests revealed key failure mechanisms, including fiber pull-out, matrix cracking, and delamination, which are influenced by the fiber orientations. Future work will include additional number of ply inclusion and the surface treatment of the fibers to improve the fiber-matrix adhesion through oxygenation using chemical treatment. Surface treatment can improve the surface energy, which may lead to better bonding and improved mechanical property. This study provides a baseline to explore the various options to improve the performance of the natural hybrid laminated composite for the automotive industry, a greener solution for the future.

## CRediT authorship contribution statement

**Mohammad Kamruzzaman:** Writing – original draft, Methodology, Investigation, Data curation, Conceptualization. **Samiul Alam:** Writing – review & editing, Supervision, Methodology, Investigation, Data curation.

## Declaration of generative AI and AI-assisted technologies in the writing process

During the preparation of this work the authors used ChatGPT in order to improve the readability and language of the manuscript. After using this tool, the authors reviewed and edited the content as needed and takes full responsibility for the content of the published article.

## Declaration of competing interest

The authors declare that they have no known competing financial interests or personal relationships that could have appeared to influence the work reported in this paper.
